# Posterior Ligamentum Complex Preservation Alleviate ASD‐Related Biomechanical Deterioration in Lumbar Interbody Fusion Models: A Finite Element Analysis

**DOI:** 10.1002/jsp2.70030

**Published:** 2025-01-07

**Authors:** Lipeng He, Tingchen Zhu, Weiye Cai, Wenhao Yang, Zan Chen, Jingchi Li

**Affiliations:** ^1^ Department of Orthopaedics Jiangsu CM Clinical Innovation Center of Degenerative Bone & Joint Disease, Wuxi TCM Hospital Affiliated to Nanjing University of chinese Medicine Wuxi Jiangsu Province People's Republic of China; ^2^ Department of Orthopedics, Luzhou Key Laboratory of Orthopedic Disorders, The Affiliated Traditional Chinese Medicine Hospital Southwest Medical University Luzhou Sichuan Province People's Republic of China; ^3^ Department of Orthopedic, The Affiliated Hospital Southwest Medical University Luzhou Sichuan Province People's Republic of China

**Keywords:** biomechanical deterioration, capsule, iatrogenic capsule injury, motion segment, posterior lumbar interbody fusion

## Abstract

**Background:**

There are differences in the extent of excision of articular processes, spinal processes and posterior ligamentum complexes (PLC) for posterior approach lumbar interbody fusion. Given that the biomechanical significance of these structures has been verified and that deterioration of the biomechanical environment is the main trigger for complications in both fused and adjacent motion segments, changes in decompression ranges may affect the potential risk of adjacent segmental disease (ASD) biomechanically; however, this topic has yet to be identified.

**Methods:**

Posterior lumbar interbody fusion (PLIF) with different decompression strategies was simulated in a well‐validated lumbosacral model. The excision and preservation of the cranial motion of the segmental PLC and the lateral articular process in the fusion segment were simulated in this model. The stress distribution in the cranial motion segment was computed under different loading conditions to determine the potential risk of ASD.

**Results:**

Compared to complete bilateral articular process excision, preservation of the lateral two‐thirds of the articular process did not alleviate stress concentration on the cranial motion segment both in PLC preserved and excised models. In contrast, preservation of the cranial segmental PLC can obviously alleviate the stress concentration tendency of the cranial intervertebral disc under flexion loading conditions.

**Conclusion:**

Preservation of the lateral parts of the articular process cannot optimize the biomechanical environment, in contrast, PLC preservation can effectively alleviate ASD related biomechanical deterioration of the cranium segment.

## Graphical abstract

(1) Changes in decompression ranges affect the potential risk of ASD biomechanically. (2) Preservation of PLC can alleviate the risk of cranial motion segmental biomechanical deterioration. (3) PLC preservation in PLIF operation can reduce the risk of ASD biomechanically.

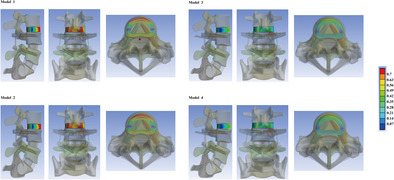

## 1 Introduction

Bilateral pedicle screw fixed posterior lumbar interbody fusion (PLIF) can effectively achieve wide‐range nerve decompression and credible segmental stability reconstruction in the fusion segment and has been widely used in patients with lumbar degenerative diseases (LDD) [1, 2]. Adjacent segment disease (ASD) is a typical complication in patients who undergo surgery via the PLIF [3, 4]. Biomechanical deterioration in adjacent segments, especially in the cranial motion segment, is the initial trigger for ASD [4, 5]. Therefore, several surgical optimization strategies have been presented in both clinical and biomechanical studies [6, 7].

Specifically, there are still definite differences in decompression ranges due to differences in nerve structure compression positions and personal preferences of surgeons in clinical practice. Some surgeons are accustomed to complete resection of the bilateral inferior articular processes and lower two‐thirds of the laminae to achieve complete decompression of the posterior structure, but others limit the range of resection to the medial third of the articular processes. In nonfusion operations, the limited range of articular process excision can preserve the segmental stability of the operative motion segment [8, 9]. Our published studies also showed that the limit of articular process excision can also alleviate cranial adjacent segmental biomechanical deterioration during nonfusion discectomy [10, 11]. However, whether this procedure can also reduce the potential risk of ASD biomechanically in PLIF patients has yet to be identified.

Moreover, in a traditional excision of the spinous process, the surgeon removes the entire spinous process along with the posterior ligamentum complexes (PLC), which consist of both supraspinous and interspinous ligaments [12, 13], in both the fused and cranial motion segments. For example, when the L4 vertebral body is excised during the L4‐L5 PLIF operation, the attachment points of both the L4‐L5 and L3‐L4 PLCs are damaged simultaneously. In contrast, in recent years, several surgeons have attempted to excise only the lower part of the spinous process to preserve the attachment point of the cranial motion segmental PLC [13, 14]. Given that the PLC has biomechanical significance for maintaining physiological load transmission in motion segments [13, 15], PLC preservation may also alleviate the biomechanical risk of ASD in patients with PLIF operation. However, the biomechanical significance of preserving the PLC has yet to be distinctly identified in PLIF models that maintain identical decompression ranges, differing only in the excision or preservation of the cranial side spinal processes. To identify these topics and provide biomechanical references for decompression range optimization in the PLIF operation, biomechanical changes caused by excision and preservation of the articular process and PLC were simulated in our previously calibrated and validated lumbo‐sacral numerical model. To our knowledge, this was the first study to explore this topic.

## 2 Materials and Methods

### 2.1 Description of the Previously Reported Model Construction and Validation Strategy

Posterior approach simulations with different decompression ranges were performed in a lumbo‐sacral model. Model construction, pre‐processing, and simulation in this study were performed in “Solidworks 2017” and “Ansys workbench 2020 r2 academic,” respectively. The detailed model construction and validation strategy has been described in our published studies. In this modeling strategy, we perform a series of procedures to achieve an efficient balance between computational credibility and efficiency. Specifically, irregular surfaces from reconstructed models are a main source of computational error and computational burden [16, 17]. To solve this problem, the outlines of the reconstructed models are overlapped by regular outlines. By using this method, irregular surfaces of reconstructed models can be removed. In addition, the morphological parameters of bony endplates (BEPs) were defined according to the imaging data and fresh specimen measurements rather than just according to the findings for a single sample [18, 19].

Moreover, when constructing the nonbony component of the numerical model, the cross‐sectional area ratio between the nucleus and the intervertebral disc (IVD) and the ratio between the IVD and vertebral bodies were defined according to imaging measurements and model calibration [20, 21]. The zygapophyseal joint (ZJ) consists of facet cartilage and capsules [22, 23]. The capsule and different ligamentum structures were defined as the “line body” in the preprocessing process of finite element analysis [24, 25]. The intact model was calibrated by adjusting the stiffness of ligaments [26, 27]. After completing the above steps, multiple indicator model validation procedures were performed. In this process, the motility and stress parameters of the numerical model were computed under different loading conditions. The validation parameters included the range of motion (ROM) of the motion segment, intervertebral disc pressure (IDP), and disc compression value of the IVD and the facet contact force of the ZJ cartilage [28, 29, 30]. By performing the model validation procedure from different components and loading conditions, the computational credibility can be effectively verified. In this process, differences between computed values and average values from in vitro mechanical tests were compared [31, 32]. The results showed that the difference was less than one standard deviation from the in vitro test results, and this numerical model was validated well. Different sizes of hybrid mesh were utilized in the components of the numerical models for this study. In our previously published research, a mesh convergence test was conducted on the intact model. By adjusting the mesh size, IDP values were calculated under flexion loading conditions, and convergence was deemed achieved when the variation in computed IDP was less than 3% [33, 34]. The Table 1 presents the mesh sizes and material properties of various components, which are consistent with those reported in our prior studies [33, 34].

**TABLE 1:** Material properties and mesh sizes of model components.

Components	Elastic modulus (MPa)	Poisson's ratio	Cross‐section (mm^2^)	Mesh sizes (mm)
Cortical	E_xx_ = 11 300 E_yy_ = 11 300 E_zz_ = 22 000 G_xy_ = 3800 G_yz_ = 5400 G_xz_ = 5400	V_xy_ = 0.484 V_yz_ = 0.203 V_xz_ = 0.203		1.7
Cancellous	E_xx_ = 140 E_yy_ = 140 E_zz_ = 200 G_xy_ = 48.3 G_yz_ = 48.3 G_xz_ = 48.3	V_xy_ = 0.45 V_yz_ = 0.315 V_xz_ = 0.315		2.6
Bony endplates	12 000	0.3		1.2
Annulus	Hypoelastic material			1.6
Nucleus	1	0.49		1.3
Cartilage endplates and facet cartilages	10	0.4		0.7
Anterior longitudinal ligaments	Calibrated load‐deformation curved under different loading conditions	0.3	60	
Posterior longitudinal ligaments	Calibrated load‐deformation curved under different loading conditions	0.3	21	
Ligamentum flavum	Calibrated load‐deformation curved under different loading conditions	0.3	60	
Interspinous ligaments	Calibrated load‐deformation curved under different loading conditions	0.3	40	
Supraspinous ligaments	Calibrated load‐deformation curved under different loading conditions	0.3	30	
Intertransverse ligaments	Calibrated load‐deformation curved under different loading conditions	0.3	10	
Capsular	7.5 (< 25%) 32.9 (> 25%)	0.3	67.5	
PEEK OLIF Cage	3500	0.3		1.5
Titanium alloy screw and connection rod	110 000	0.3		1.2

### 2.2 Simulation of Posterior Approach Lumbar Interbody Fusion With Different Decompression Ranges

As mentioned above, there are obvious differences in the extent of decompression used for posterior approach lumbar interbody fusion. Consistent with the findings of published studies, the L4–L5 motion segment was selected for surgical procedure simulation because it has the highest incidence of LDD [34, 35]. Therefore, fusion operations were simulated for different decompression ranges according to the literature review and our clinical experience. Specifically, in the numerical model with complete excision of posterior structures, including the spinous process, attach‐point of PLC, lower two‐thirds of the laminae, and whole articular process on both sides of the L4 vertebral body were excised (Model 1) [3, 36]. Moreover, to simulate an operation involving limited articular process excision, the lateral two‐thirds of the inferior articular process of the L4 vertebral body were preserved (Model 2). Finally, when simulating the preservation of the cranial segmental PLC, the cranial part of the spinal process was preserved based on Model 1 and Model 2 (Table 2).

**TABLE| 2:** Variations in excision ranges across different numerical models.

	Cranial part of spinal process	Bilateral lamina and articular processes
Model 1	Excision	Excision
Model 2	Excision	Preservation
Model 3	Preservation	Preservation
Model 4	Preservation	Excision

**FIGURE 1:**
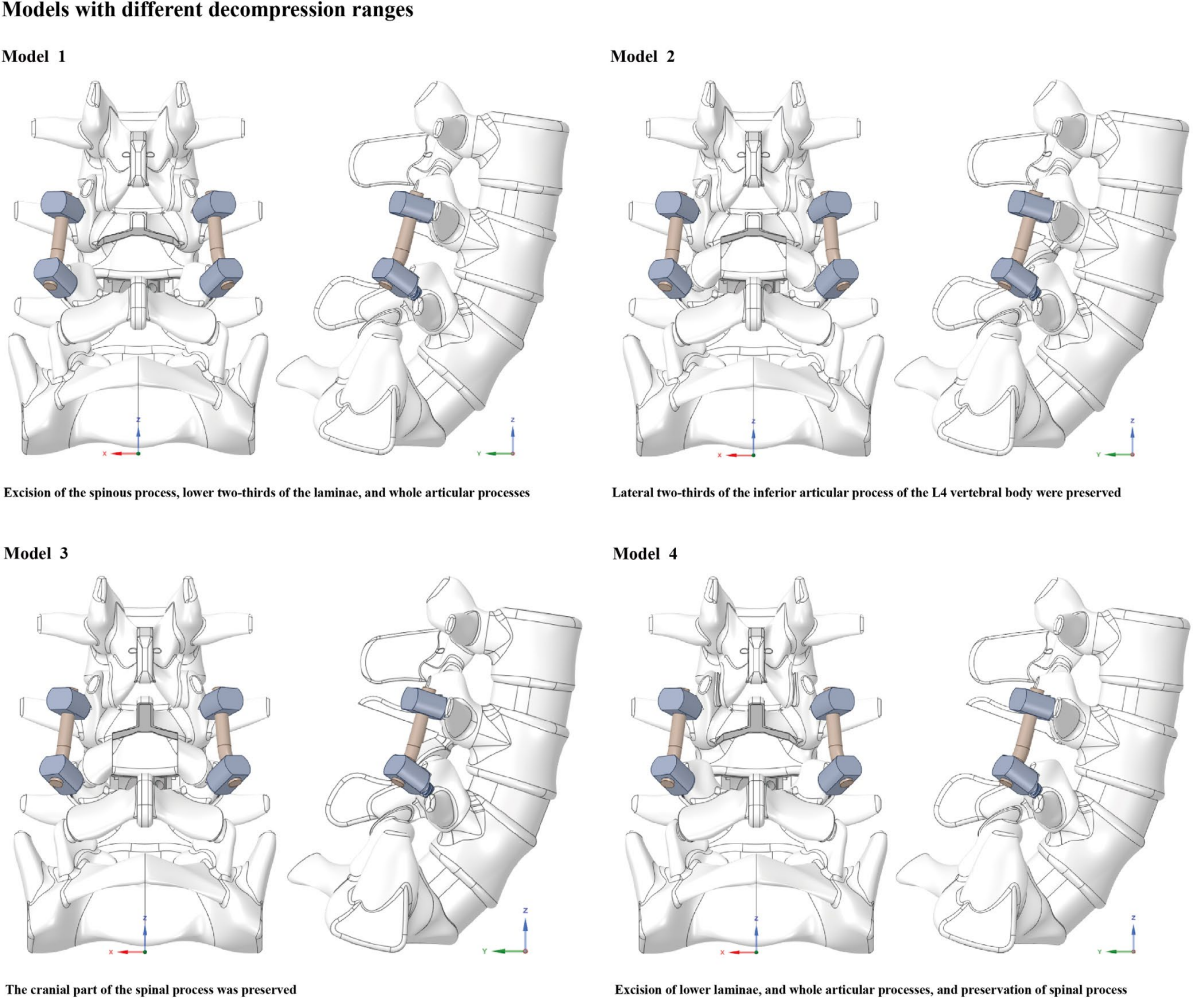
Construction strategies for L4‐L5 PLIF models: Model 1: Completed excision of articular processes and the L4 spinal process; Model 2: Preservation of the lateral two‐thirds of the articular process and complete excision of the L4 spinal process; Model 3: Preservation of the lateral two‐thirds of the articular process and the superior part of the L4 spinal process; Model 4: Excision of articular processes and preservation of the L4 spinal process.

The simulation of interbody fusion and pedicle fixation procedures was performed according to published studies (Figure 1). Specifically, to simulate bilateral pedicle screw fixation and interbody fusion, pedicle screws were inserted into both the L4 and L5 vertebral bodies. When constructing pedicle screw models, the screw tulip and nut were simplified to a simple structure to optimize the computational efficiency [34, 37]. The diameter of pedicle screw and connection rod were 6.5 mm and 6.0 mm, respectively, and the screw length was 45 mm. According to the literature review, the screw trajectory was parallel to the superior BEP of the vertebral body on the sagittal plane and parallel to the axis of the pedicle on the transverse plane [38, 39]. Moreover, to simulate bony compaction caused by screw insertion, the elastic modulus of bony structures around the screw trajectory was adjusted [40, 41, 42]. The range of adjusted bony structures consisted of the volume of the screw. Given that different grades of thread insertion may affect the local biomechanical environment, posterior bony structures around the screw trajectory were excised so that threads could be completely inserted into the vertebral bodies [37, 42]. Cartilage endplates and all nuclei were excised from the fused IVD (L4‐L5 IVD). When simulating cage insertion, a cage (26 mm length, 10 mm wide, and the height and contour of the cranial and caudal surfaces were aligned with the outline of the BEP) was inserted into the interbody space from the right side of the interbody space [34, 37].

### 2.3 Boundary and Loading Conditions

**FIGURE 2:**
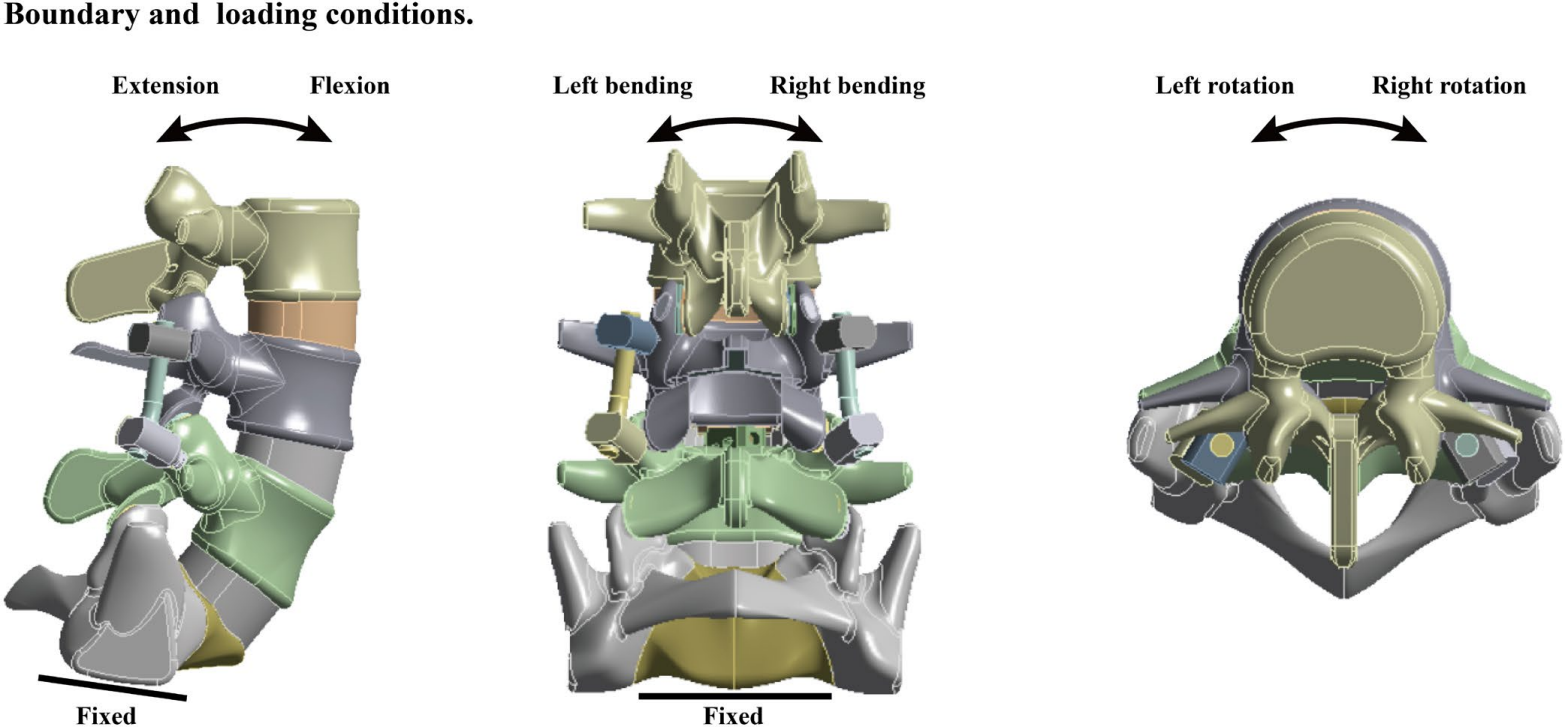
Boundary and loading conditions for the current models: The inferior surface of the models was completely fixed, and different directional moments were applied on the superior surface of L3. These boundary and loading conditions can be used to simulate in vitro mechanical tests effectively.

The caudal side of the S1 was completely fixed at all degrees of freedom. Different directional moments were applied on the superior surface of L3. The moment sizes changed in different directions: those of flexion, extension, bending, and rotation were 8, 6, 6, and 4 Nm, respectively (Figure 2) [26, 43]. The loading conditions utilized during the stress computation process were consistent with those employed in model validation [26, 27], this computational strategy enhances the credibility of our results. When defining the contact types, the friction coefficient between the bone‐screw interfaces and cage‐bone interfaces was established at 0.2 [34, 37]. In contrast, the friction coefficient between the grafted bone and BEP was determined to be 0.46 [34, 37]. All other interface contact types were classified as “bonded” [33, 34].

Given that the PLIF procedure is most frequently performed in the L4‐L5 motion segment, which demonstrates the highest incidence of LDD [44, 45], it is noteworthy that, compared with the caudal side, the cranial motion segment experiences a significantly elevated occurrence of ASD [46, 47]. Furthermore, variations in the range of spinal process excision may only influence the preservation or removal of the cranial segmental PLC attachment point. As a result, this study has thoroughly analyzed and documented the biomechanical environment within the L3‐L4 motion segment.

## 3 Results

**FIGURE 3:**
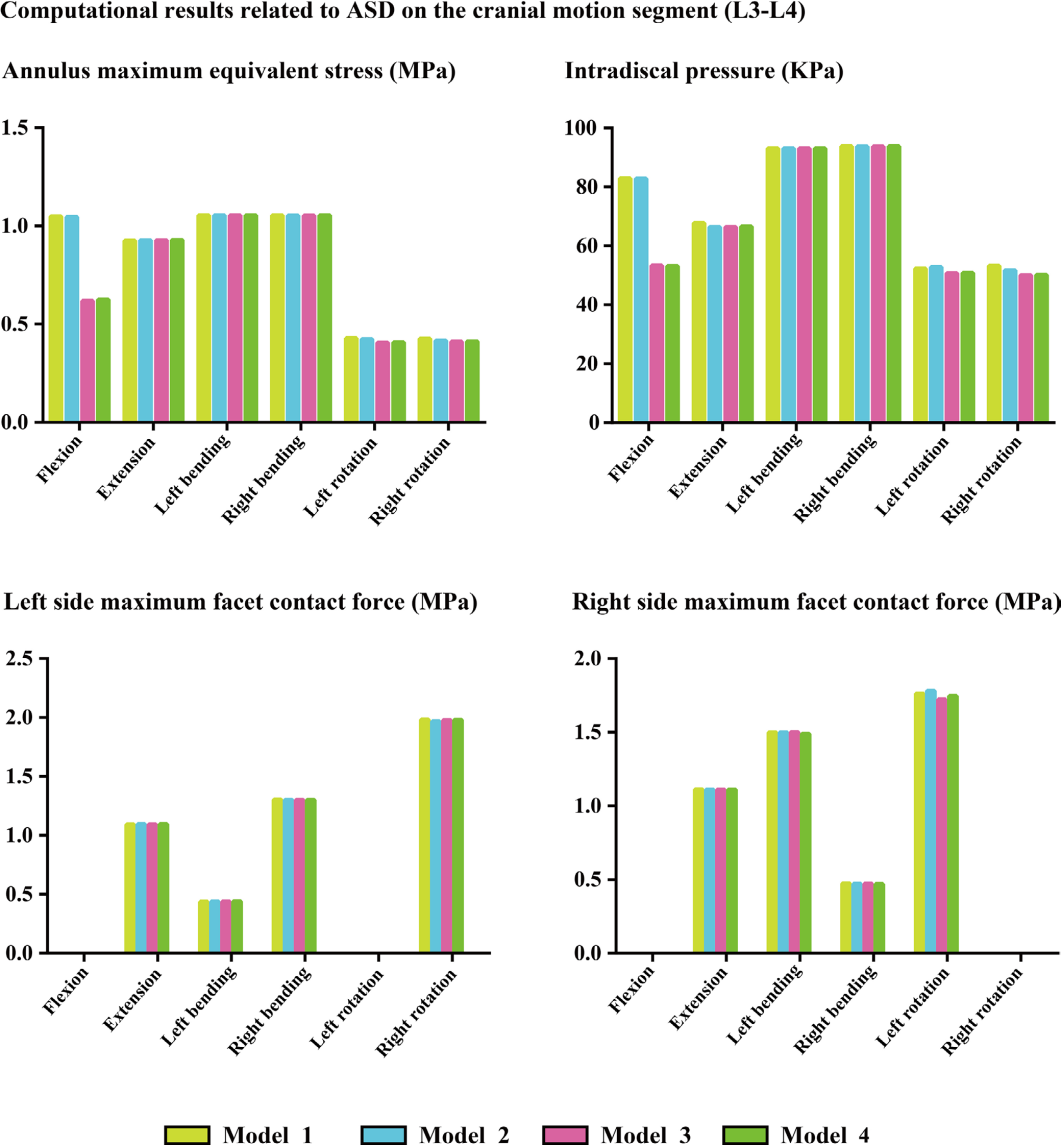
Computed results: Compared to those in Model 1 and Model 2, the stress concentration in the IVD was effectively alleviated in the Model 3.

**FIGURE 4:**
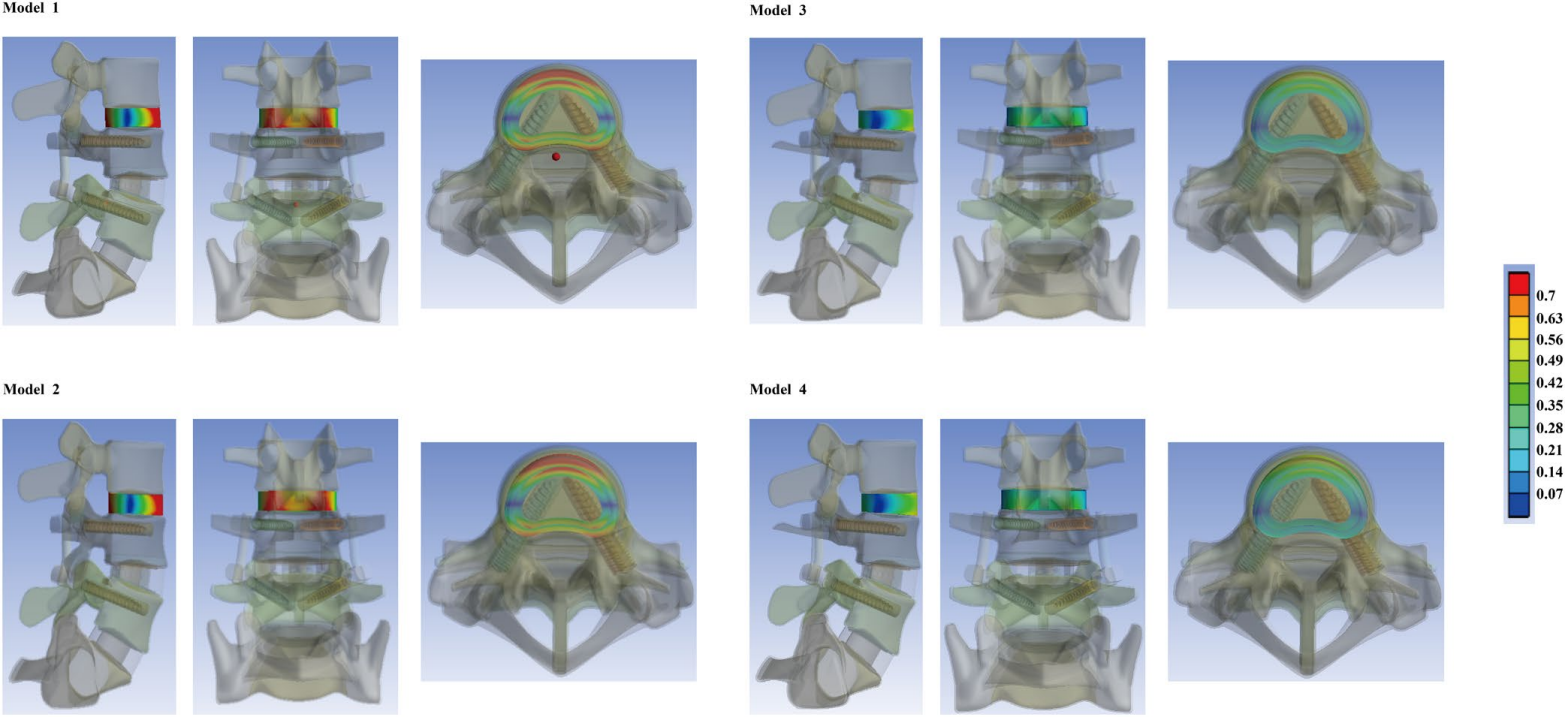
Nephograms of the stress distribution in the annulus under flexion loading conditions: Comparable stress values can be recorded in both Models 1 and Model 2, and a lower stress value can be observed in Model 3 and Model 4. Therefore, biomechanical significance of articular processes excision and preservation was limited. In contrast, obvious difference in stress values on the cranial motion segment can be observed between models with PLC excision and preservation.

The maximum stress on the annulus, IDP, and the maximum stress on the facet cartilages were calculated and documented to assess the potential risk of ASD in the L3‐L4 motion segment. The biomechanical significance regarding the cranial motion segment following excision versus preservation of the articular process was minimal in both models with PLC excision and preservation. The differences in computed values between Model 1 and Model 2 were less than 3% under nearly all loading conditions; a similar trend was also observed between Model 3 and Model 4. In contrast, a notable decrease in stress was observed in the IVD under flexion loading conditions in models with preservation of the PLC. Specifically, compared to models exhibiting iatrogenic PLC damage (Model 1 and Model 2), the maximum stress on the annulus was reduced by over 30%. While that on IDP decreased by more than 40% under flexion loading conditions in models where cranial spinal process preservation was maintained (Model 3 and Model 4). Furthermore, both excision and preservation of the PLC and articular processes did not obviously affect stress distribution across cranial segmental facet cartilages. All computational results along with corresponding nephograms for stress distribution are presented in Figures 3 and 4.

## 4 Discussion

As an operation‐related complication, ASD can trigger a deterioration in patient prognosis, and corresponding revision surgery is also an important cause of social and economic burdens [5, 48]. Biomechanical deterioration can trigger the progression of ASD, and the biomechanical mechanism of surgical optimization strategies has been presented in published studies [49, 50]. For instance, compensatory stress concentration in the segments adjacent to the fused vertebral body represents a fundamental pathological mechanism underlying the progression of ASD [5, 46]. Biomechanical research indicates that longer fusion segments can exacerbate this compensatory stress concentration at adjacent levels [5, 49]. Clinical reviews have further established this factor as a significant contributor to the increased incidence of ASD [46, 47]. Additionally, iatrogenic injury to the cranial side articular processes has been associated with a higher occurrence of ASD [46, 47]; biomechanical studies demonstrate that such injuries can induce segmental instability and accelerate degenerative changes [51, 52]. Consequently, recognizing the potential biomechanical implications on adjacent segments resulting from surgical interventions can offer valuable theoretical guidance for optimizing surgical strategies.

Given that the articular process and spinal process (especially the attached PLC) have inherent biomechanical significance [14, 15], preservation of these structures may reduce the risk of ASD biomechanically. In this study. A PLIF operation involving iatrogenic injury and preservation (or partial preservation) of the PLC and articular processes was performed, and stress values related to ASD were computed and recorded in the cranial motion segment (L3‐L4). According to published studies, PLC, as a typical type of tension band, can effectively maintain segmental stability under flexion loading conditions [14, 53]. In comparison to these studies, significantly lower stress values are observed in models where the PLC is preserved during flexion loading. Given that elevated annulus stress and IDP can lead to a higher incidence of annular tears and accelerate disc degeneration, preserving the PLC during PLIF may effectively mitigate the potential biomechanical risks associated with ASD.

Although the biomechanical significance of PLC preservation have been investigated in published studies [53, 54], surgeons compares adjacent segmental biomechanical performance in models involving unilateral lamina and articular process excision, as well as bilateral excision of lamina and articular, and the spinal processes. In other words, this study did not investigate the biomechanical significance of the spinal process and articular processes independently; it failed to elucidate any confounding effects caused by other lateral articular processes. In contrast, our current study identifies the distinct biomechanical effects of both the spinal process (i.e., PLC) and articular process on cranial adjacent segments separately. This represents a significant innovation point of our research.

The following topics should be clarified from a methodological perspective. First, only stress values in the cranial motion segment were computed in this study. Although ASD on the caudal side still exists, epidemiological studies have recorded a significantly greater incidence of ASD in the cranial motion segment in PLIF patients [39, 50]. From a biomechanical perspective, pedicle screw fixation induces high stiffness in the surgical segment, and the resulting adjacent segmental stress concentration and motility compensation trigger the generation of an ASD [55, 56]. Given that the distance between the cranial pedicle screw and the cranial adjacent motion segment was significantly shorter than that of the caudal segment, more dramatic biomechanical deterioration and a greater incidence of ASD were observed in the cranial motion segment [39, 47]. More significantly, during PLIF, the spinal process of the cranial vertebral body, rather than the caudal body, is excised. Therefore, the PLC on the caudal side adjacent to the motion segment will not be damaged. (e.g., when the L4‐L5 PLIF was completely excised, the attachment point of L3‐L4 was injured, but that of the L5‐S1 PLC was kept intact.) Therefore, biomechanical deterioration caused by iatrogenic injury to the PLC can be observed only in the cranial motion segment, and these reasons determine that the biomechanical environment of the cranial motion segment was computed in this study.

In addition, although our published studies showed that a limited range of articular process excision can reduce the risk of ASD biomechanically, the current study showed that a larger range of articular process excision did not trigger biomechanical deterioration of the cranial adjacent motion segment in patients who underwent PLIF. Differences in computed results may be rooted in differences in the main triggers of biomechanical changes between fusion and nonfusion operations. Specifically, in patients who underwent nonfusion spinal surgery (e.g., discectomy), changes in excision range can affect not only the surgical but also the motility of adjacent segments [50, 57]. In contrast, pedicle screw fixation plays a primary role in biomechanical changes, and the biomechanical significance of these changes in the excision range was limited [58, 59]. Therefore, a larger range of articular process excision was recommended based on the current computational results when necessary.

In contrast, although spinal process excision was performed in the fusion segment, the attachment point of the cranial segmental PLC was damaged during this procedure. In other words, minimizing the destruction of the physiological structure of adjacent segments was an effective method for reducing the risk of ASD biomechanically. Finally, although only observable differences can be recorded in flexion loading conditions, we still believe that PLC preservation can alleviate the risk of ASD. Even if the patient can be instructed to avoid lumbar flexion movement during the early postoperative period, it is difficult to strictly eliminate this movement during daily life. As a result, microdamage accumulation during this period can trigger the progression of ASD, and this topic was also validated by the same type of study [12, 52].

Admittedly, the lack of clinical evidence was the main limitation of this study. Partial excision of the spinal process is an innovative surgical optimization strategy that we recently proposed. As a middle‐ to long‐term complication, the pathological period of ASD was more than 5 years. Therefore, we did not have enough follow‐up data to validate the clinical effect of PLC preservation. However, given that biomechanical changes can affect the risk of ASD, this study provides an important basis for carrying out subsequent clinical research.

## 5 Conclusion

By performing a numerical mechanical simulation in a well‐validated lumbosacral model, this study revealed that preservation of the lateral parts of the articular process cannot optimize the biomechanical environment, in contrast, PLC preservation can effectively alleviate ASD related biomechanical deterioration of the cranium segment. The currently biomechanical research conclusion still should be validated by our future clinical studies.

## Author Contributions

Conception and design: Lipeng He, Zan Chen, and Jingchi Li. Model construction and finite element analysis: Lipeng He, and Jingchi Li. Analysis and interpretation of data: Tingchen Zhu, Weiye Cai, and Wenhao Yang. Figures preparation: Lipeng He and Zan Chen. Manuscript preparation: Lipeng He, Tingchen Zhu, and Jingchi Li. Manuscript modification: Zan Chen and Jingchi Li.

## Ethics Statement

The authors have nothing to report.

## Consent

The authors have nothing to report.

## Conflicts of Interest

The authors declare no conflicts of interest.

## Data Availability

All the data of the manuscript are presented in the paper.
